# Learning the Relationship between the Primary Structure of HIV Envelope Glycoproteins and Neutralization Activity of Particular Antibodies by Using Artificial Neural Networks

**DOI:** 10.3390/ijms17101710

**Published:** 2016-10-11

**Authors:** Cătălin Buiu, Mihai V. Putz, Speranta Avram

**Affiliations:** 1 Department of Automatic Control and Systems Engineering, Faculty of Automatic Control and Computers, Politehnica University of Bucharest, Bucharest 060042, Romania; 2 Laboratory of Structural and Computational Physical-Chemistry for Nanosciences and QSAR, Biology-Chemistry Department, Faculty of Chemistry-Biology-Geography, West University of Timisoara, Timisoara 300115, Romania; 3 Laboratory of Renewable Energies-Photovoltaics, R&D National Institute for Electrochemistry and Condensed Matter, Timisoara 300569, Romania; 4 Department of Anatomy, Animal Physiology and Biophysics, Faculty of Biology, University of Bucharest, Bucharest 050095, Romania; speranta.avram@gmail.com

**Keywords:** HIV-1, glycoproteins, antibodies, neutralization data, artificial neural network, regression

## Abstract

The dependency between the primary structure of HIV envelope glycoproteins (ENV) and the neutralization data for given antibodies is very complicated and depends on a large number of factors, such as the binding affinity of a given antibody for a given ENV protein, and the intrinsic infection kinetics of the viral strain. This paper presents a first approach to learning these dependencies using an artificial feedforward neural network which is trained to learn from experimental data. The results presented here demonstrate that the trained neural network is able to generalize on new viral strains and to predict reliable values of neutralizing activities of given antibodies against HIV-1.

## 1. Introduction

**Figure 1:**
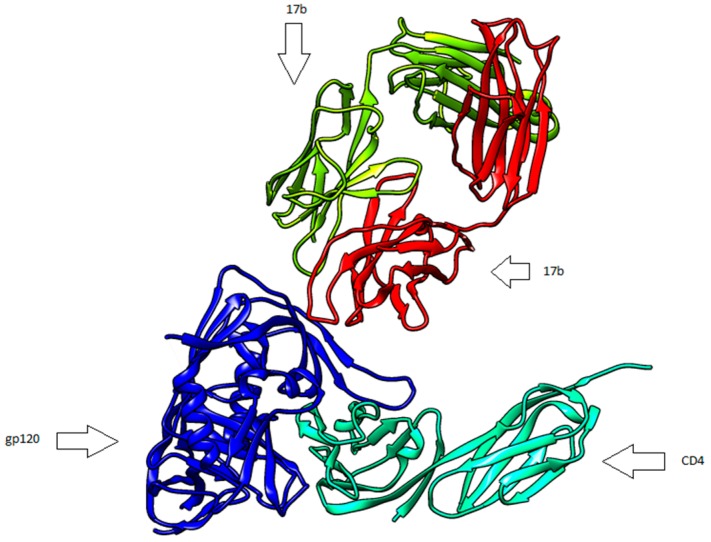
gp120 glycoprotein in complex with CD4 and an antibody (17b) (Protein Data Bank entry 1GC1).

HIV-1 entry into target cells is mediated by envelope glycoprotein (ENV) trimers [1]. ENV is a viral protein serving to form the viral envelope, and the glycosylated envelope trimer is synthesized as gp160, a precursor protein which is further cleaved by furin into gp120 and gp41 subunits [2]. A viral spike shows three gp120 glycoproteins which are noncovalent to three gp41 transmembrane molecules [3]. A key step in the viral entry is the binding of this complex to the CD4 receptor on the cell surface. Figure 1 shows the gp120 core (blue) complexed with CD4 (green) and 17b (red and yellow), which is a neutralizing human antibody.

The ENV protein is organized into five conserved regions, namely C1–C5 and five variable regions, namely V1–V5 [4]. It was reported that the exposed surface of the spike is described by the variable regions of gp120 and there is also a variety of carbohydrates that help mask the surface of the protein [4]. The ENV variability, largely reported into variable loop regions V1–V5, and also sequence mutations in gp120 [4], leads to reduced interactions with specific antibodies and represents an attractive target for anti-HIV-1 treatments. From all the V1–V5 loops structures, the V4 and V5 loops are highly disordered [4] while the structure of the V3 loop is well-defined [5].

A simplified structure-based model of the V3 loop is used in [6] to model co-receptor tropism in HIV-1. Moreover, the incorporation of ENV determinants outside the V3 loop is demonstrated to be able to improve the reliability of co-receptor usage [7].

The HIV-1 protease is playing a major role in the viral replication and a study of potential protease inhibitors using a QSAR methodology was performed in [8]. A study on the molecular dynamics of the HIV-1 protease was presented in [9]. A structural and docking analysis of HIV-1 integrase and proteins of the nuclear pore complex was investigated in [10].

It is estimated that in ~20% of HIV-1-infected individuals, antibodies that neutralize diverse HIV-1 strains develop in high titers [11]. An important goal for an HIV-1 vaccine development is the identification of broadly neutralizing antibodies (bNAbs) [12,13,14]. Among the reasons for which this vaccine development is still very challenging are the unusual traits of bNAbs [15].

A number of bNAbs against HIV-1 ENV glycoproteins have been discovered [16,17,18,19]. Most of the monoclonal bNAbs target a few major sites on HIV-1 ENV [20]: the CD-4 binding site, two glycan-dependent epitopes involving the V1/V2 and V3 loops, and the membrane-proximal external region (MPER) of the transmembrane gp41 glycoprotein. For example, three bNAbs (2F5, 4E10, and 10E8) are MPER-specific as they target a fusion-intermediate conformation of gp41 [12,21]. In a recent study [22] it is shown that amino acid changes within the MPER epitope can increase the neutralization sensitivity to multiple types of bNAbs.

Partial neutralization by 10E8 was shown to be at least in part influenced by manipulating ENV glycosylation [21]. According to some studies validated by [12], 10E8 is neutralizing HIV-1 with potency and breadth much larger than those of 2F5 and 4E10. Both 2F5 and the m66 antibodies are considered to be the only effective human HIV-1-neutralizing antibodies to recognize the N-terminal region of the MPER of the gp41 subunit of ENV. A crystal structure of m66 in complex with its gp41 epitope is presented in [23]. Antibody accessible sites in the V1–V2 domain of HIV-1 gp120 are the object of several studies, e.g., [24].

A comparison of the neutralization sensitivity for three periods of the epidemic (1987–1991, 1996–2000, 2006–2010) was discussed in [25] which reports that “progressive significantly enhanced resistance to neutralization was observed over calendar time, by both human sera and most of the bNAbs tested (b12, VRC01, VRC03, NIH45-46^G54W^, PG9, PG16, PGT121, PGT128, PGT145)”. However, a combination of NIH45-46 and PGT128 antibodies was shown to still efficiently neutralize the most contemporary transmitted variants. This analysis is extended to some recently described bNAbs (PG9-iMab, PG16-iMab, 10E8, 3BNC117, NIH45-46m2, NIH45-46m7, 10-1074, JM4sdAb, 8ANC195, and PG9-16-RSH) in [20].

As pointed out in [26,27,28], the variation of the neutralization data with respect to various HIV-1 strains is a complicated, unknown function of the ENV primary structure. There are some factors behind this complicated relationship, such as the binding affinity of the antibody to the ENV protein and the intrinsic infection kinetics of the viral strain. An intense effort is carried out in order to identify critical residues of ENV which affect antibody activity. For example, a computational tool to help identify these critical resides is presented in [28] and it is based on the simplifying assumption that the variation of neutralization activities (characterized by IC_50_ values, the concentration at which infectivity is reduced by 50% [29]) is due to amino acid identity or glycosylation state at a small number of sites, each acting independently.

This paper presents the first results of a novel approach, which is based on using machine learning, to extend this analysis of the variation of the neutralization data. Machine learning is a subfield of computer science dedicated to the development and study of algorithms that can learn from and make predictions on data [30]. A more formal definition is provided in [31]: “A computer program is said to learn from experience E with respect to some class of tasks T and performance measure P, if its performance at tasks in T, as measured by P, improves with experience E”. Artificial neural networks (ANN) are prominent machine learning algorithms which are inspired by the structure and functioning of biological neural networks. There are numerous applications of ANNs in biology and medicine, such as dihedral angles prediction in enzyme loops [32], affinity prediction of protein-ligand complexes [33], cancer prognosis and prediction [34] and computational drug development [35,36,37,38].

What we present in this paper is a preliminary work to learn the dependencies between ENV primary structures (amino acids sequences) and neutralization activity of particular antibodies. This is done by training a feedforward ANN with input data (whole ENV primary structures) and output data (neutralization values for particular antibodies) to provide a neural network which is able to generalize on other ENV glycoproteins and to predict neutralization data.

## 2. Results

A trial-and-error approach has been chosen in order to find the most suitable neural network architecture and parameters for learning the available data, as there are no universally valid guidelines for designing a neural network [39]. For this purpose, a number of experiments have been designed in which some parameters were modified in order to evaluate their impact on the network’s performance in learning and generalization.

**Figure 2:**
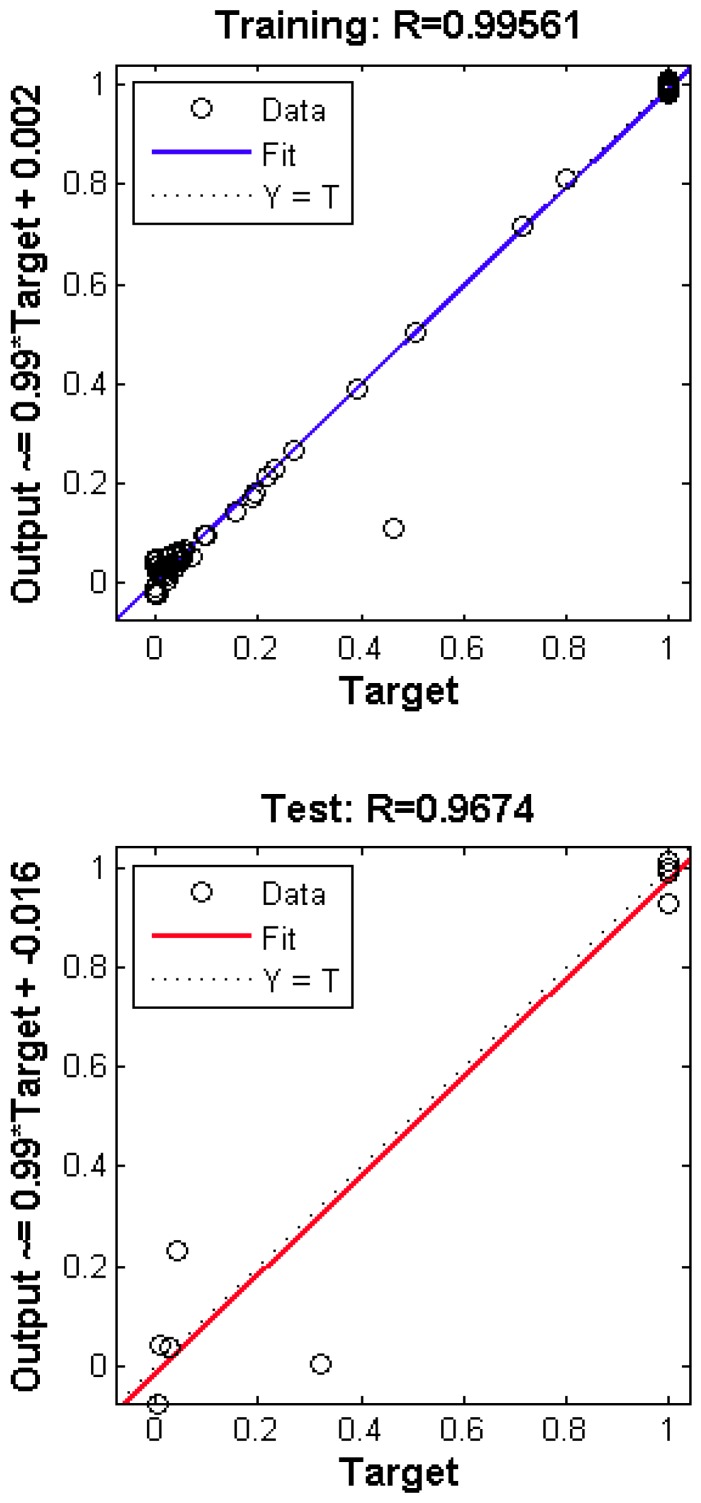
Regression analysis for the training and test data.

**Figure 3:**
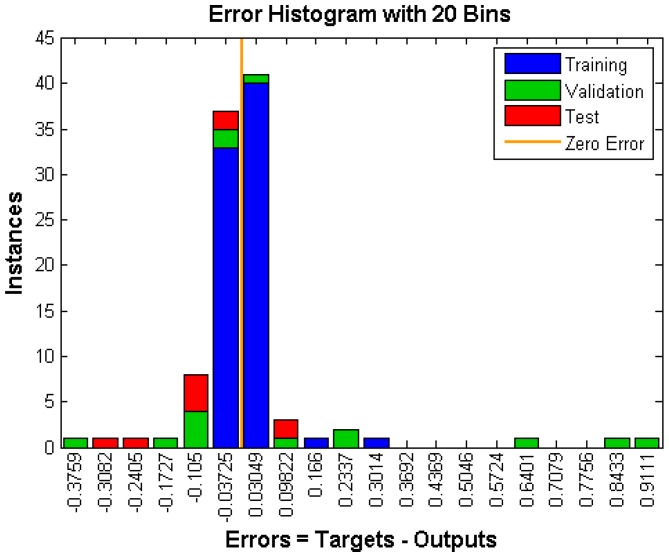
Error histogram.

First, the available data was divided randomly into training, validation and test sets. The MATLAB default values for the sizes of the three datasets were used (75%, 15%, and 10%, respectively). For the data in the training set, a Levenberg–Marquardt backpropagation algorithm [40,41] was used. Monitoring the error on the validation set allows an early stopping of the training, as overfitting is associated with a rise of the validation error. If this error increases for a specified number of iterations, then the training process is stopped. Secondly, each backpropagation training algorithm starts with different initial parameters (weights and biases), so that very different solutions can be obtained with each new training process. Thus, we repeated the above process 100 times and the network with the best generalization was selected. The statistical power of the method was evaluated by the correlation coefficient R. The results for learning the neutralization data for the 2F5 antibody show that suitable correlation coefficients were obtained for the training set (*R* = 0.99561 and dependent variable *Y* = 0.99 × Target + 0.002) and for the test set (*R* = 0.9674 and dependent variable *Y* = 0.99 × Target + 0.016). These results are shown in Figure 2 which presents the corresponding regression analysis, while Figure 3 shows the error histogram. The mean squared prediction error (MSEP) was 0.015.

## 3. Discussion

The complexity of HIV-1 ENV structural biology asks for complementary information obtained from various techniques such as NMR spectroscopy, X-ray crystallography, cryo-electron microscopy or tomography to understand the virus infectious mechanism, but the limitations of each of these technologies are evident [4]. Given the limitations of each of these approaches, the challenge for the future HIV-1 ENV studies may be represented by in silico methods (e.g., chemical structures-biological activity relationship) for structural biologists in the HIV field to aim higher.

The work presented in this paper is based on our expertise in studying the chemical structures-biological activity relationship HIV-1 protease by using ANNs [42] and also chemical structures-biological activity relationship HIV-1 gp120 in interaction with different antibodies [43]. In [43] we calculated the pharmalogical descriptors of the HIV-1 gp 120 binding sites structures for 60 HIV-1 strains. We considered steric molecular descriptors (molecular surfaces, volumes), electronic descriptors (electrostatic energies), counts of atoms and bonds types (number of atoms, number of hydrogen donors or acceptors and number of rigid bonds). We identified: (1) the possible correlation between molecular descriptors of HIV-1 gp 120 and their biological activities; (2) significant fluctuation of descriptors among the strains. Also in [42], we used ANNs to evaluate the biological activity of HIV-1 protease inhibitors for QSAR-like applications and we found that the local mapping of ligand properties, applied to HIV-1 protease, provides accurate results (95%).

This paper presents a novel approach in trying to predict antibody affinities from a primary HIV-1 ENV sequence using a trained feedforward neural network. This has been demonstrated to be an efficient tool to learn dependencies between HIV-1 envelope glycoproteins’ primary structure and neutralization activities for particular antibodies. This paper introduced both the idea and the practical realization of a way to model IC_50_ neutralization data variation across a panel of HIV-1 strains.

Results demonstrate that a carefully trained network can learn the nonlinear and complicated dependencies between ENV primary structures and neutralization data for particular antibodies. Partial Least Squares (PLS) regression is widely used in chemometrics [44] for relating two data matrices by a linear multivariate model. We used the Statistics and Machine Learning Toolbox in Matlab in order to relate the input data (aligned ENV sequences) to output data (neutralization data for a particular antibody, 2F5 in our case).

**Figure 4:**
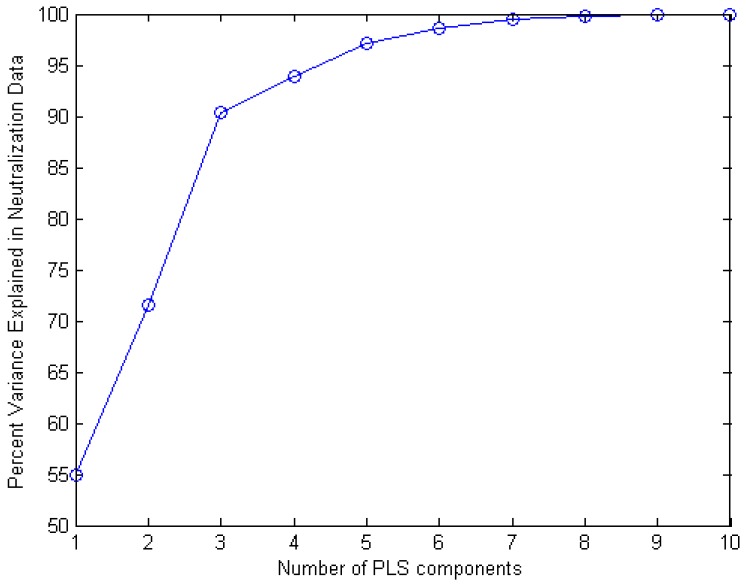
Percent of variance explained in the response variable as a function of the number of Partial Least Squares (PLS) components.

The first step was to fit a PLS regression model with ten PLS components and one response. We generated and analyzed the percent of variance explained in the response variable as a function of the number of components. Figure 4 shows that ten components fully explain the variance.

**Figure 5:**
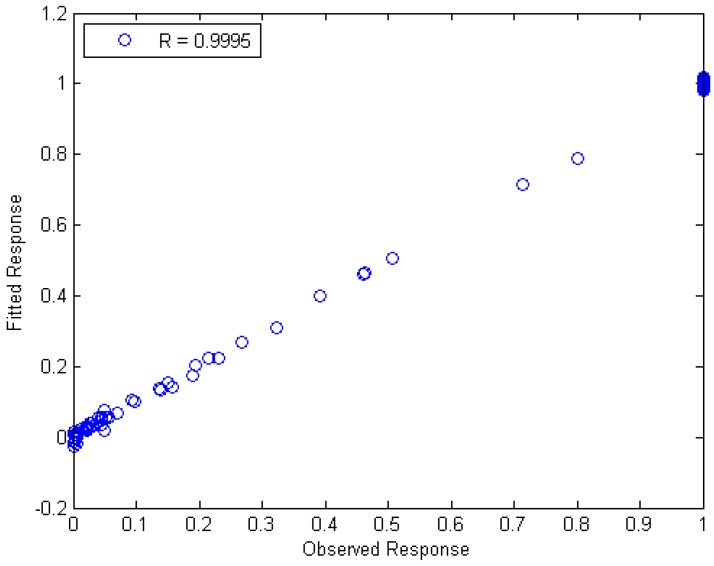
Fitted response vs. observed response for the Partial Least Squares (PLS) regression.

Figure 5 then shows the fitted response vs. the observed response for the PLS regression with ten components with *R* = 0.9995.

**Figure 6:**
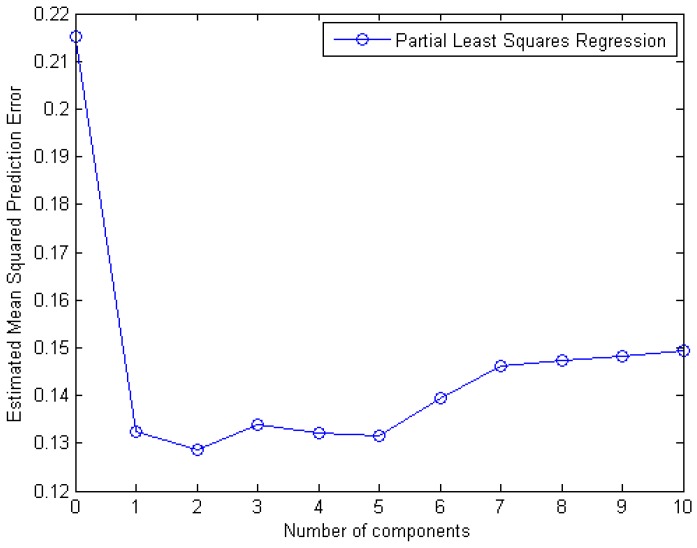
Mean squared prediction error as a function of the number of Partial Least Squares Regression components.

A ten-fold cross-validation technique was then used for estimating the mean squared prediction error (MSEP) which is 0.15 as it can be seen in Figure 6.

So, the neural network based approach has generated an MSEP ten times smaller than the Partial Least Squares regression.

In this preliminary study, our results improve the knowledge about the HIV-1 ENV protein, its molecular and possible neutralization properties. This ANN-based method can be applied on a large number of HIV-1 ENV structures with large variability. The trained neural network is able to generalize and to predict neutralization data for particular antibodies across HIV-1 strains which were not included in the training set.

Future work will include the acquisition of more neutralization data, and more aligned ENV sequences. Particular attention will be paid to the study of the influence of the glycosylation sites and amount of glycosylation. A sensitivity analysis will be implemented for the trained network, in order to determine which inputs (which residues of ENV glycoproteins) affect the output (neutralization activity) most. This sensitivity analysis can be implemented based on two methods: a backward stepwise method in which one variable (ENV residue) is blocked (rejected), and the effect on the output is quantified; and a second weight method which is based on the weights magnitude. This sensitivity analysis will suggest critical residues as candidates for mutagenesis studies.

A better understanding of the biological activity of HIV-1 ENV structures can be achieved by performing both experimental and in silico studies and we will focus our next studies in this direction. We are sure that, in the near future, our study can be extended by experimental techniques which are able to explore more precisely the molecular features of HIV-1 ENV structures. Even though the biological processes in HIV-1 ENV structures involved are very complex and difficult to replicate in vivo, the extension of our study by in vivo analyses is crucial.

## 4. Materials and Methods

The goal of the research reported here was to find an implicit model for the relationship between the primary structure of HIV-1 ENV proteins and neutralization data (IC_50_ values (µg/mL)). All the programs were written in Matlab^TM^ (R2012A) and the Matlab’s Neural Networks Toolbox (version 7.0.3, MathWorks^®^, Natick, MA, USA) was used. Microsoft Excel was used for archiving neutralization data. The experiments were run on a computer with an Intel(R) Core(TM) i7-3160QM CPU @ 2.30 GHz, 16 GB installed memory and a 64-bit operating system.

The critical importance of ENV regions variability for the HIV-1 infective process and also for the virus escape from antibody interactions was already mentioned. Our aim in these preliminary experiments was to predict, in the most accurate way possible, the interactions of a large number of HIV-1 ENV strains.

The input sequences (primary structures for various HIV-1 strains) can vary in length. As the input of the neural network is fixed in length, our approach was to use aligned sequences. Other possible approaches were to use sparse-encoding [45] or interpolation [46].

Aligned ENV sequences (input data for our network) were collected from the HIV Sequence Database (http://www.hiv.lanl.gov/) [47] which we used for downloading ENV data in a FASTA file which contains 4907 aligned ENV sequences. These ENV alignments are based on the complete genome nucleotide alignment. So, the input data for our approach is represented by global alignments of ENV proteins from a large number of HIV-1 strains. The length of the global alignment is 1369.

The output (neutralization) data was collected from literature [16,17,19,29,48,49,50,51,52] and stored in Microsoft Excel files where each row corresponds to a different viral strain and each column corresponds to a different antibody, e.g., 2F5, VRC01, NIH45-46, 3BNC117, PG9, and PG16. Data in these files is represented by IC_50_ (the half maximal inhibitory concentration) values and where the IC_50_ for a particular case is known only to be greater than or less than some value (e.g., 50 µg/mL), then that specific value was selected. The FASTA file and a sample Excel file with neutralization data (178 HIV-1 strains and neutralization data for six antibodies) are publicly available for download [53].

**Figure 7:**
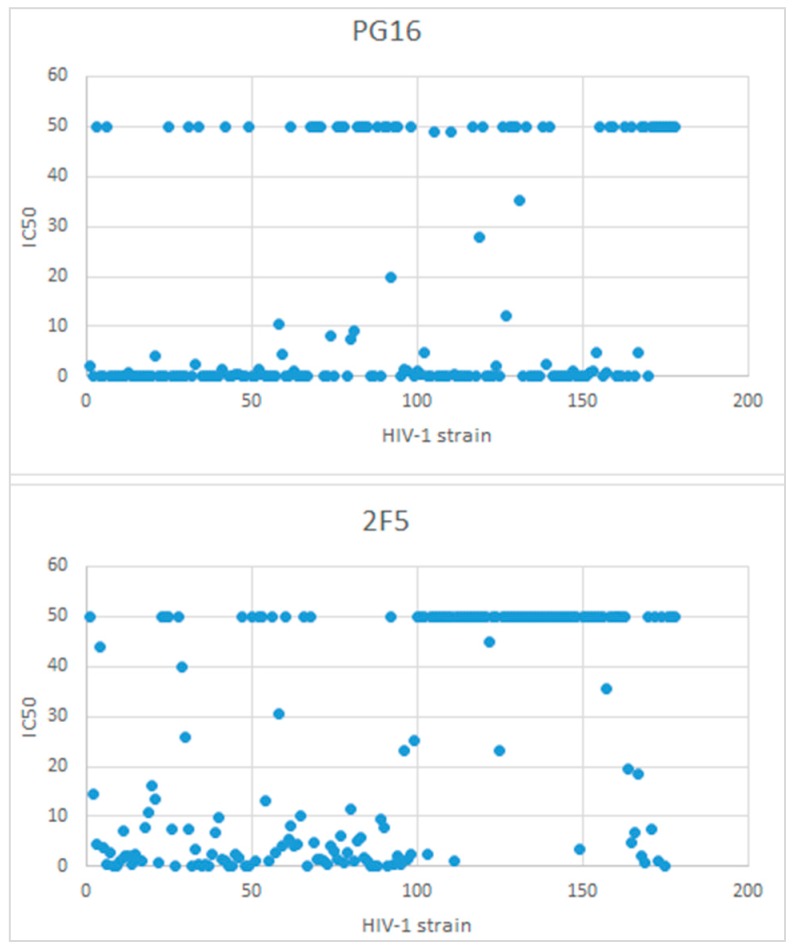
Distribution of IC_50_ values for selected antibodies (PG16 above, and 2F5 below) against all the 178 strains in the sample file.

The sample neutralization data looks like in Table 1, while Figure 7 shows the distribution of the IC_50_ values for two antibodies (PG16 and 2F5) against the 178 HIV-1 strains: the distribution of the majority of values around 50 µg/mL and below 1 µg/mL for PG16, while for 2F5 the values are more scattered between 0 and 50 µg/mL.

**Figure 8:**
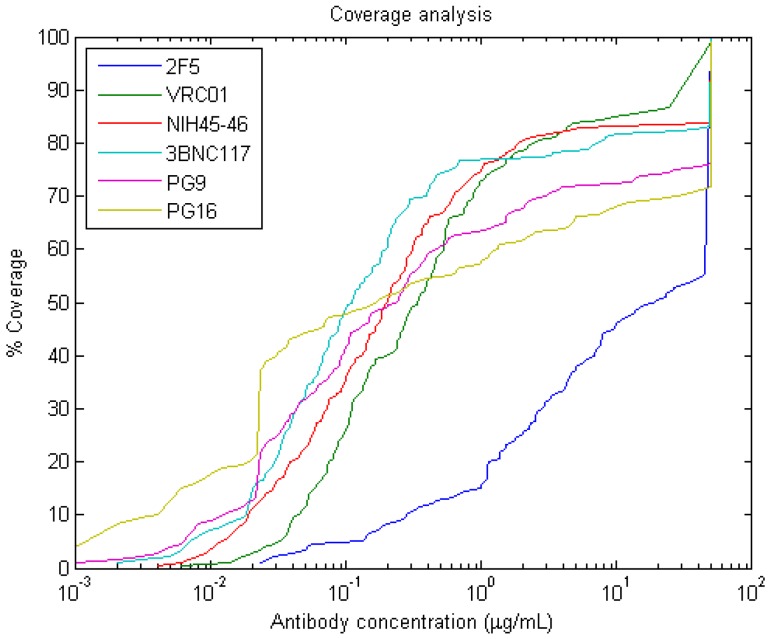
Coverage curves for given antibodies.

These data files are read into MATLAB and further used for training the ANN. A coverage curve can be generated for these antibodies using a Matlab function we designed, in order to compare neutralization across a panel of HIV-1 strains. In Figure 8, coverage curves were generated for the data in the sample file (a coverage curve shows the cumulative frequencies of IC_50_ values up to the concentration which is shown on the *x* axis [13]).

Variations of these neutralization values against different strains are complicated functions of ENV sequences, as noted in the introduction. Our idea was to model these complicated dependencies using the powerful learning capabilities of a feedforward neural network trained to minimize the error between the target and actual neutralization data and, further on, to use the generalization abilities of these networks to predict IC_50_ values for different strains.

**Figure 9:**
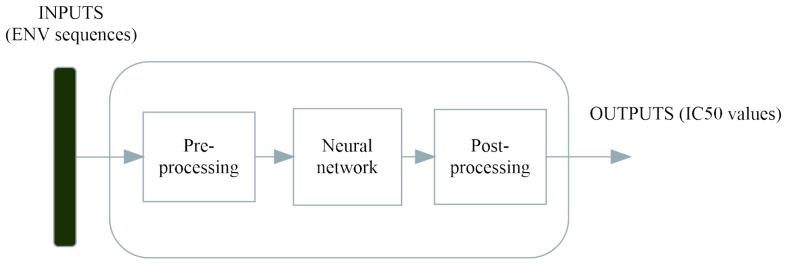
Modeling (learning) neutralization data using a feedforward neural network.

In Figure 9, we present the generic structure of our neural network as a function approximator between inputs (ENV primary structures) and outputs (IC_50_ values).

It is known that such a neural network may learn, in appropriate conditions, any nonlinear relationship between input and output data. The overall process has a number of steps which are detailed in the following for our specific application.

### 4.1. Collecting Data

Input (ENV sequences)/output (neutralization values) data is collected as indicated above.

### 4.2. Creating the Network

This step is about creating a Matlab neural network object using the predefined *fitnet* function which produces a feedforward neural network whose parameters will be specified during the next steps.

### 4.3. Configure the Network

**Figure 10:**
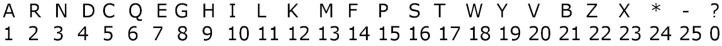
Correspondences between amino acids and integers in our codification scheme.

At this step, we specify the number of inputs, number of hidden layer neurons, and number of outputs. As the input vectors are aligned ENV sequences of a length of 1369 characters, these 1369 positions are provided to the input neurons. The network can be fed with other aligned ENV sequences, and if the length of the alignment is different, then the number of input nodes will change accordingly. The input data has to be converted to a numeric format. This is done in a simple way by using the correspondence table in Figure 10, where B is D or N (aspartic), Z is E or Q (glutamic), X represents any amino acid, * represents an end terminator, - is a gap, and ? is an unknown amino acid. This mapping is the amino acid letter codes to integers coding used in the Bioinformatics Toolbox from Matlab.

The network will have one output for to the IC_50_ value corresponding to a particular antibody, e.g., 2F5. The number of neurons in the hidden layer is adjustable (the implicit value is 10). The output data is normalized to (0,1).

### 4.4. Initializing the Network

This is accomplished by generating random values for the network’s weights and biases.

### 4.5. Training the Network

The goal is to learn, in the best possible way, the input–output relationship which is implicit in the ENV primary structure—neutralization data dependency. Training an artificial neural network generally means finding the optimal values for the network’s weights and biases in order to minimize a performance index *F*, which is usually the mean square error (*mse*) as indicated below:

(1) $$F=mse=\frac{1}{N}\overset{N}{\underset{i=1}{\sum }}{\left(e_{i}\right)}^{2}=\frac{1}{N}\overset{N}{\underset{i=1}{\sum }}{\left(t_{i}-a_{i}\right)}^{2}$$

where *N* is the number of input–output pairs, *t* (for target) is the desired output (experimental IC_50_) of the network and *a* (for actual) is the actual output of the network.

Regularization is usually used for improving the generalization abilities of the neural network. In this case, the performance function (1) above is modified to:

(2) msereg=γ×msw+(1−γ)×mse

where:

(3) $$msw=\frac{1}{n}\overset{n}{\underset{i=1}{\sum }}w_{j}^{2}$$

and *w* are the network’s weights, and *γ* is the performance ratio (usually 0.5).

The standard feedforward network training is based on the Levenberg–Marquardt algorithm [40], and other widely used alternatives are Bayesian regularization and BFGS quasi-Newton methods.

### 4.6. Validating the Network

After training has been finished, one has to check the network’s performance. This can be done by checking the training record (an output of the training process together with the trained network) in order to see if changes with regards to training procedure, networks architecture and parameters, are needed. Dynamic values for the performance index and the gradient are also available from the training record. The next step is the generation of a regression plot (network response vs. corresponding targets), which provides a view of the dependency between the desired output and the actual output of the network. It is also possible to plot the error histogram plot which shows the distribution of the network errors.

### 4.7. Utilizing the Network

As previously indicated, the utility of having such a network available is the possibility to provide any new ENV sequence at its input and thus to predict the neutralization data for the modeled antibody (one of the six antibodies in our case: 2F5, VRC01, NIH45-46, 3BNC117, PG9 and PG16).

## Figures and Tables

**Table 1:** Sample data used for training the network (HIV-1 strains in the first column, neutralization data for six selected antibodies in the other columns).

HIV-1 Strain	2F5	VRC01	NIH45-46	3BNC117	PG9	PG16
0260.v5.c36	50	0.529	0.397	0.2	2.18	2.1
0330.v4.c3	14.6	0.064	0.049	0.013	0.018	0.006
0439.v5.c1	4.43	0.052	0.185	0.215	50	50
3415.v1.c1	43.9	0.092	0.082	0.094	0.149	0.036
3718.v3.c11	3.88	0.218	0.871	50	0.05	0.019
398-F1_F6_20	0.28	0.058	0.157	0.071	50	50
BB201.B42	2.92	0.343	0.303	3.35	0.014	0.003
BB539.2B13	0.136	0.094	0.022	0.033	0.106	0.012
BI369.9A	0.249	0.149	0.043	0.02	0.029	0.007
BS208.B1	1.1	0.029	0.006	0.002	0.031	0.004
KER2008.12	6.98	0.563	0.567	0.248	0.017	0.006
KER2018.11	2.01	0.07	0.828	0.417	0.001	0.001
KNH1209.18	2.24	0.087	0.246	0.04	0.367	0.678
MB201.A1	0.436	0.237	0.165	0.464	0.024	0.001
MB539.2B7	2.49	0.544	0.402	0.087	0.058	0.025
MI369.A5	1.44	0.162	0.074	0.033	0.058	0.011
MS208.A1	1.1	0.147	0.09	0.019	0.071	0.047
Q168.a2	7.83	0.14	0.138	0.05	0.106	0.031
Q23.17	10.8	0.086	0.106	0.017	0.007	0.002
Q259.17	16.1	0.051	0.046	0.017	0.045	0.028
Q461.e2	13.4	0.41	0.212	0.069	3.01	4.11
Q769.d22	0.609	0.015	0.013	0.007	0.007	0.01
Q769.h5	50	0.014	0.019	0.006	0.002	0.002
Q842.d12	50	0.006	0.015	0.002	0.005	0.001
QH209.14M.A2	50	0.024	0.011	0.008	50	50
RW020.2	7.55	0.303	0.144	0.02	0.103	0.07
UG037.8	0.202	0.035	0.056	0.02	0.021	0.001
3301.V1.C24	50	0.084	0.055	0.046	0.281	0.023
6540.v4.c1	40	50	50	50	0.035	0.017
6545.V4.C1	26	50	50	50	0.095	0.068
0815.V3.C3	7.37	0.036	0.055	0.018	50	50
6095.V1.C10	0.147	0.464	0.601	0.096	0.242	0.023
3468.V1.C12	3.51	0.04	0.104	0.073	2.09	2.38
620345.c1	0.455	50	50	50	0.393	50
C1080.c3	0.056	1.5	0.539	0.096	0.004	0.001
C2101.c1	0.344	0.097	2.38	0.064	0.026	0.009
